# The role of narrative in collaborative reasoning and intelligence analysis: A case study

**DOI:** 10.1371/journal.pone.0226981

**Published:** 2020-01-06

**Authors:** Morgan Saletta, Ariel Kruger, Tamar Primoratz, Ashley Barnett, Tim van Gelder, Robert E. Horn

**Affiliations:** 1 SWARM Project, University of Melbourne, Melbourne, Victoria, Australia; 2 Human Science and Technology Advanced Research Institute (H-STAR), Stanford University, Stanford, California, United States of America; King’s College London, UNITED KINGDOM

## Abstract

This paper explores the significance of narrative in collaborative reasoning using a qualitative case study of two teams of intelligence analysts who took part in an exercise using an online collaborative platform. Digital ethnographic methods were used to analyze the chat transcripts of analysts as they reasoned with evidence provided in a difficult, fictional intelligence-type problem and produced a final intelligence report. These chat transcripts provided a powerful “microscope” into the reasoning processes and interactions involved in complex, collaborative reasoning. We found that Individuals and teams used narrative to solve the kinds of complex problems organizations and intelligence agencies face daily. We observed that team members generated what we term “micro-narratives”, which provided a means for testing, assessing and weighing alternative hypotheses through mental simulation in the context of collaborative reasoning. The creation of micro-narratives assisted in the teams’ reasoning with evidence, an integral part of collaborative reasoning and intelligence analysis. Micro-narratives were combined into, and compared with, an ideal or ‘virtual’ narrative which informed the judgements the team came to in their final intelligence report. The case study developed in this paper provides evidence that narrative thought processes play an important role in complex collaborative problem-solving, reasoning with evidence and problem-solving. This is contrary to a widespread perception that narrative thinking is fundamentally distinct from formal, logical reasoning.

## Introduction

Decision-makers and analysts are often faced with complex, uncertain and rapidly evolving problems and situations where data and information may be incomplete and where judgements and decisions have serious, at times even life-and-death ramifications. These problems and situations may, for example, relate to how best to organize emergency disaster relief after a major earthquake, and whether a given building houses a terror mastermind such as Osama bin Laden.

There has been a widespread perception, following Jerome Bruner [1], that narrative thinking is fundamentally different to formal, logical and scientific thinking (e.g. Dahlstrom [2]). Over the past few decades, research within fields ranging from jurisprudence and criminology to human evolution has examined the role of narrative in thinking and reasoning [3–6].

We think that narrative reasoning plays a crucial and underappreciated role in complex problem-solving. This includes the context of intelligence analysis. However, while Clark [7] and Heuer and Pherson [8] discuss the role of narrative in producing scenarios for strategic planning, and the National Academy of Sciences Decadal Survey highlights the importance to intelligence analysts of narratives, in the press, social media and society there has been scant attention to the role of narrative *reasoning* in intelligence analysis, despite Gregory Treverton’s [9] suggestion that “…intelligence is ultimately about telling stories.”

One area of complex decision-making where the role of narrative reasoning has been extensively analyzed is the area of jurisprudence [4, 10, 11]. According to the story model of jury decision-making, story construction plays a critical role in the way jurors evaluate evidence and make verdict decisions. We think that narrative reasoning and story construction is an essential part of complex reasoning, problem-solving and knowledge-building, and this beyond the context of the existing story model of jury decision-making.

In this paper we develop insights into the role of narrative in collaborative reasoning and intelligence analysis through a case study in which two teams of analysts from an Australian Commonwealth Government agency or department with intelligence functions worked on a fictional but realistic intelligence-type problem using an online collaborative platform developed by the SWARM project at the University of Melbourne. The SWARM project aims to develop fundamental advances in collaborative reasoning and knowledge-building as part of the Crowdsourcing Evidence, Argumentation, Thinking and Evaluation (CREATE) program launched by the U.S. Intelligence Advanced Research Projects Activity (IARPA) in 2016.

Online collaboration platforms such as SWARM provide opportunities for using collective intelligence [12] to transform vast amounts of data and information into knowledge [13] and to respond and coordinate responses to rapidly evolving situations such as by the U.S. Air Force’s Crisis Action Team and the 2010 Haiti Earthquake [14]. Crucially, online collaborative platforms provide new opportunities for understanding the processes involved in collaborative learning, reasoning, problem-solving and knowledge-building [15, 16].

On the SWARM platform, for example, the participants made extensive use of the in-built chat function to discuss the problem they are working on. Because these chats are saved and archived, we have access to the real-time discussion by the team members. This has given us a “microscope” with which to view and analyze the collaborative reasoning process. In the exercise, we observed that participating teams of intelligence analysts used stories to make judgements based on a complex problem and uncertain information. Team members using the chat function of the online SWARM platform generated numerous examples of “micro-narratives”, corresponding to what Vlek et al [17] term ‘subscenarios’ or ‘substories’. As reasoning using stories has been observed in other domains [4, 18], we believe that the phenomenon we have observed on the SWARM platform is not limited to the SWARM or other collaborative platforms—rather, the SWARM platform has enabled this process to be recorded and observed in a new way, and has given us novel insights into the narrative reasoning process.

We observed in the chat transcripts that the analysts generated a multitude of micro-narratives in their discussions of the evidence and their assessment of the problem characters’ involvement in terrorist activities. We infer that these micro-narratives analysts were telling each other are verbal representations of stories about the evidence that the analysts were telling themselves in their own minds, as they imagined various alternative ‘hypotheses’ to explain the evidence.

We thus follow Raymond Mar, Keith Oatley and others [19–22] in theorizing that narratives are mental simulations that help people to understand the world and others. We infer from our analysis of the chat transcripts that team members “ran” mental simulations to test and evaluate many diverse, interacting and often competing micro-narratives that were generated collaboratively with other team members using the chat function of the SWARM platform. The analysts thus tested the plausibility of these micro-narratives against each other, what they knew, and their best estimates (or guesses) for what they didn’t know.

In a non-linear and iterative process, the analysts used the insights developed in the process of generating and evaluating micro-narratives to develop a macro-level narrative. Micro-narratives contribute to this macro-level narrative but are also compared to it for fit, plausibility, and so on. This macro-level narrative was never fully written down, but we inferred its existence from the chat transcripts and from the final reports, which contain elements of this larger narrative. We refer to this macro-level narrative as ideal, in the sense that it existed largely in the minds of the team members. We also refer to it as ‘virtual’ in the sense that the narratives were, in our view, mental simulations that helped the team members reason about evidence in a complex problem.

Narrative is generally defined as a description of real or fictional events, situations, actions, etc. in a time-sequence [23]. While it might seem that evidence is already naturally connected by a narrative structure, this is not typically the case in jurisprudence [4] or in intelligence analysis. Data and evidence are presented or available in an often disconnected and even fragmentary form. In the intelligence-type problems given to teams, evidence, intelligence and relevant information to the problem took the form of statements summarizing interviews with witnesses, as well as forensic lab reports and background reading material for understanding the use of PCR in forensic investigations. Additionally, each team member naturally began the exercise possessing their own background knowledge, assumptions and generalizations about the world, much of which is tacit knowledge [24]. All these disparate sources of information about the world and about the problem at hand must be brought together into a coherent structure. We think that narrative offers a powerful and natural means of doing this, but observed that connecting this information into a coherent plausible narrative or narratives took considerable effort.

## Materials and methods

### The SWARM platform

SWARM is a cloud-based platform that allows teams to collaborate on a single, coherent, comprehensively reasoned and argued intelligence report. The SWARM project has conducted a series of experiments and exercises in which teams use the SWARM platform to collaboratively solve complex problems and produce collaboratively written reports which are the subject of ongoing quantitative and qualitative analysis. One of our broad research goals is to understand how teams reason and produce knowledge products in pursuit of the SWARM project’s overarching goal of producing fundamental improvements in collaborative reasoning and knowledge-building in intelligence analysis [25, 26].

**Fig 1:**
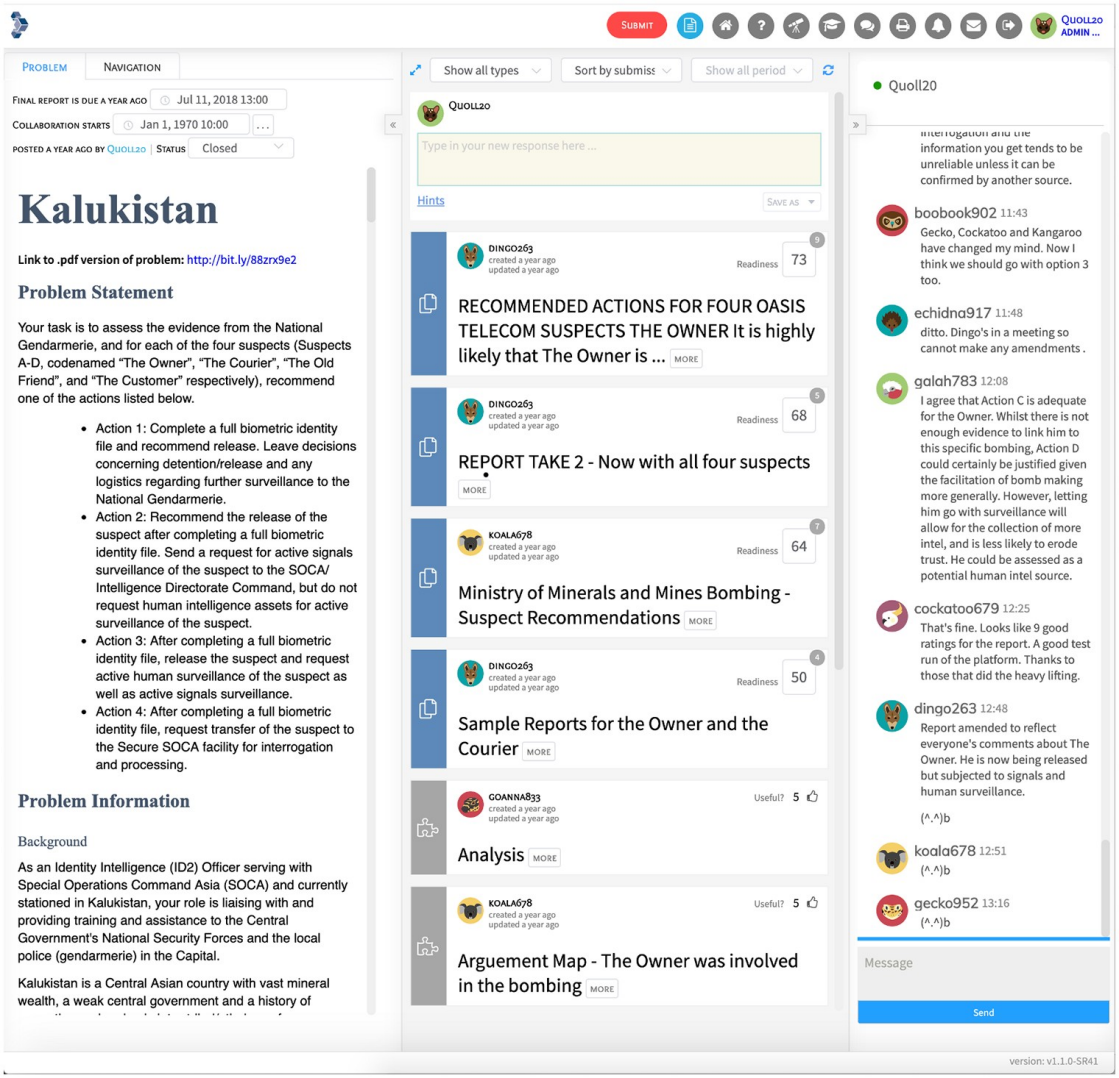
The Swarm platform. The workspace is divided into a problem pane (left), a work pane (middle)—where team members draft their reports and post resources, and a chat pane (right).

The SWARM platform is similar in some ways to familiar commercial products such as Google Classroom, but also provides a ‘lens kit’ of logical lenses, or cognitive tools for thinking about and solving intelligence problems. These include Structured Analytic Techniques (SATs) such as link analysis, alternative hypotheses and the SWARM project’s own contending analyses, techniques for statistical thinking, and other tools for thinking to help users in their reasoning and analysis. See Fig 1 for a sample image of the SWARM platform.

### Participants

In January 2018, an Australian Commonwealth government agency or department with intelligence functions opted to take part in a study facilitated by the SWARM project and carried out on the SWARM platform. Participation was offered to intelligence analysts in the final stages of their training. At the study’s commencement, 34 anonymous participants had consented to take part. The 34 participants were divided into two independent teams of 17, codenamed “Purnululu” and “Kakadu” (SWARM teams are named after Australian place names). The study received ethics approval from the University of Melbourne’s Faculty of Science Human Ethics Advisory Group. All participants were over the age of 18, were given a plain language statement and digitally signed an informed consent statement.

### Materials

#### The “Kalukistan Problem”

In our case study, we presented the two participating teams with a fictional but realistic intelligence-style problem akin to a problem that professional analysts might encounter. The problem was presented to the two teams on the SWARM platform, a screenshot of which is included below. Each team collaborated on an analysis and on writing a report addressing the intelligence-type problem.

The “Kalukistan Problem” is set in a hypothetical Central Asian country where conflict and terrorism are rife. The teams assumed the role of an intelligence officer working for “SOCA”, a Western intelligence agency which has recently issued a memorandum for its intelligence officers setting out constraints that the officer’s decisions must abide by. According to the problem statement, an important aspect of the officer’s work is described as assessing when further information might assist a given investigation, and how to prioritize that information-gathering, which means taking into account the resources required to gather the information and any other relevant constraints. Recently there has been a bombing which targeted Westerners at the Ministry of Minerals and Mines headquarters, in connection with which four suspects have been apprehended. The intelligence officer’s immediate task is to recommend, based on a range of data provided, actions regarding continued detention and questioning or the release and/or continued observation of each suspect.

The problem has two main parts. The first step is to clarify the decision-making procedure. Given the possible actions available and a memorandum from SOCA concerning interrogation and surveillance protocols, the officer must determine how exactly to judge what action is appropriate in a given case. The second main part of the problem is to assess, for each suspect, the likelihood of his or her involvement in terrorist activity, given the data currently in the officer’s possession. How does the officer do this rigorously and systematically, resulting in clear and well-reasoned probability judgements? This is particularly challenging given that the evidence is incomplete and inconclusive. This is deliberate, and the problem is designed such that good analytical reasoning requires more than *ad hoc* consideration of the evidence and "gut feel" analysis. Team members were required to collaborate to produce a final co-created report on the SWARM platform with analysis of and reasoning about the provided data leading to recommended actions for each of the four suspects.

### Research approach

This case study is part of ongoing research carried out within the larger SWARM project. A fundamental question driving this research is, how do individuals and teams reason in order to come to a solution to an intelligence-type problem?

While this ongoing research is informed by digital ethnographic, or netnographic [27], methodologies, and by Edwin Hutchins’ study of cognitive processes in real-world settings [28–30], the case study in this paper is limited to a qualitative, thematic analysis [31, 32] of the online chat transcripts and final reports generated on the SWARM platform in the aforementioned exercise run in 2018 with analysts from an Australian Commonwealth Government agency or department with intelligence functions.

#### Stage 1—Exploratory sense-making

The first stage of the process was to read the transcripts and final reports in order to get a sense of how each team employed reasoning in order to arrive at a solution. Authors 1 and 2 engaged in an initial round of exploratory coding. During this initial exploratory coding and sense-making exercise, author 6 identified that participants in the study were proposing short, fragmentary and conjectural stories—micro-narratives—using the chat function. Subsequent thematic analysis suggested that the teams then evaluated these micro-narratives by comparing them against other micro-narratives with respect to the evidence and intelligence presented in the problem statement, and their relationship to the events and evidence presented in the problem. Teams also evaluated and compared the way micro-narratives could be combined and integrated together into a larger virtual narrative that they had started to develop in their minds. These virtual, or macro-narratives, were never fully written down or recounted in full, but we could infer their existence from the data in the chat transcripts and final reports. The approach we employed in this research was iterative and recursive, and rather than provide a richly detailed description of the entire data set, we chose to produce a detailed account of the theme ‘Micro-narratives’.

#### Stage 2—Exploratory coding of micro-narratives

Using the NVivo 12 software, Authors 1 and 2 then conducted a thematic coding [33] of these short stories that were being proposed by participants in the chat transcripts; Author 1 coded the Kakadu team and Author 2 coded Purnululu. Without a rigid definition of what a ‘micro-narrative’ is or how to reliably identify micro-narratives, the best coding approach was to propose a definition wide enough in scope to include all potential micro-narratives before refining it in view of the data. The definition used—*a sentence or passage of text*, *submitted by a member of the team*, *that describes an action(s) or sequence of events—* was informed by the exploratory sense-making stage of the process and possesses an appropriate scope for capturing all—but not only—the text of interest:

The initial exploration of the transcripts suggested that the definition above needed refinement, as it included passages regarding commentary, judgements about team tasks, and discussion of evidence. For example, the following chat is a commentary on the individual’s progress and only tangentially related to the model of interest:

13:43 Kookaburra31: Started working on my initial thoughts, but mainly writing all of my info gaps!
13:43 Quokka37: Yep—initial thoughts. Nothing crazy, quick for and against for each suspect with some other thoughts.
13:44 Kangaroo24: Yea I’m just writing down some initial thoughts, thinking about hypotheses and assumptions in particular before thoughts on suspects

#### Stage 3—Final thematic coding for micro-narratives and initial theorization

A new definition was then proposed that worked to exclude the unwanted types of chat messages described above:

We understand micro-narratives as short, often fragmentary descriptions of states of mind (such as motiviations or intentions), actions, reactions, consequences, sequences or sets of events that a person or persons (intelligence analysts in our study) use to reason with evidence, make judgements and reach conclusions.

Authors 1, 2 and 3, using NVivo 12, independently undertook a round of thematic coding of the two transcripts for micro-narratives. The goal was to compare each author’s results with all the others’ to ensure that the definition was reliably identifying passages of text as micro-narratives. In their coding of Purnululu, authors 1 and 3 scored an agreement of 89.06% according to NVivo, and in their coding of Kakadu’s chat, authors 2 and 3 achieved an agreement of 86.07%. These results confirm that the authors agree ~85–90% of the time on what portions of the transcript could be appropriately labelled ‘micro-narratives’. This provides a good level of confidence that the thematic coding was picking up a genuine phenomenon in the text.

#### Stage 4—Further understanding and theorizing the role of micro-narratives

In the final stage, we turned our attention to better understanding and attempting to theorize the role of micro-narratives in the overall reasoning of the teams and how this came to be reflected in the intelligence reports they were tasked with creating.

At the beginning of this stage, we had a preliminary model for thinking about how micro-narratives were being used in the collaborative reasoning process, and how they were being used to create the final intelligence reports. Author 3 conducted a further round of coding, focusing on different modes of collaborative work—that is, conversation fulfilling different roles within the team’s thinking and knowledge-product creation process—that occurred in the chat transcript and were marked by shifts, or transitions, in its conversation, in order to understand the overall structure of the reasoning process and the role micro-narratives play within it.

**Fig 2:**
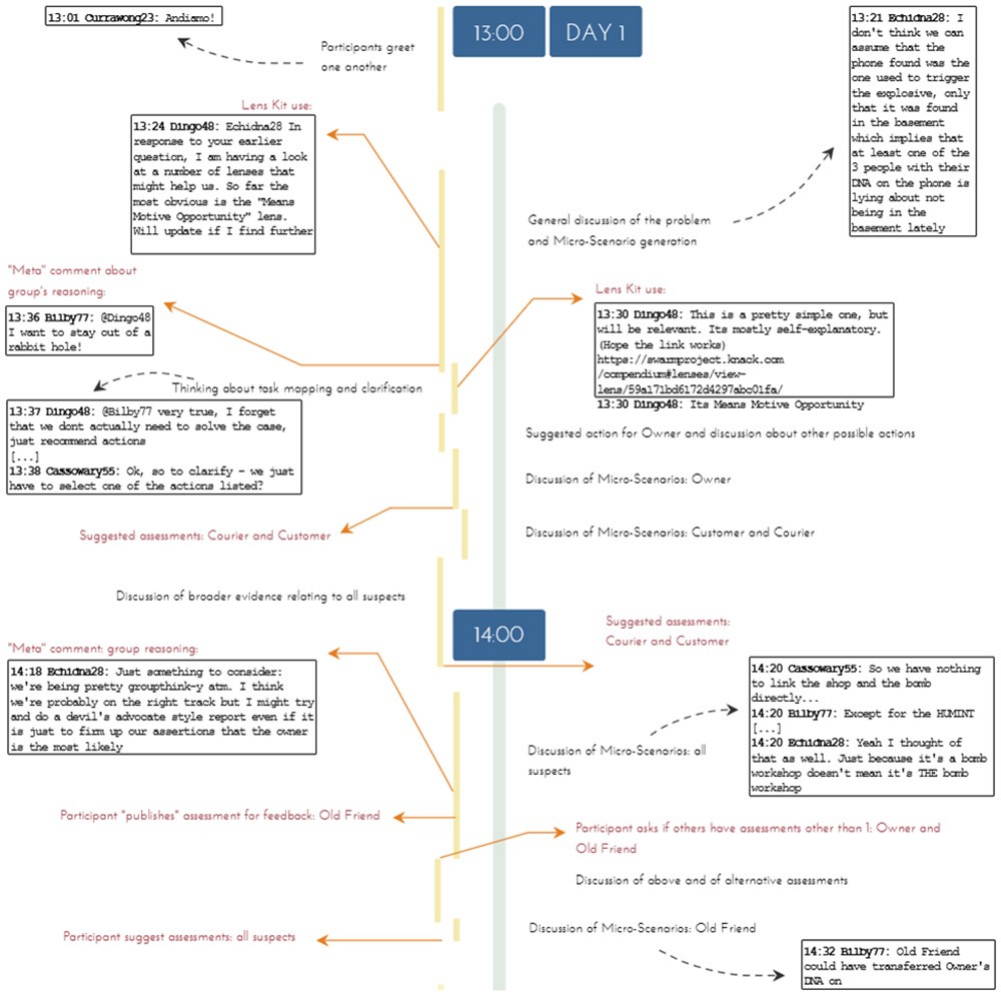
A sample selection of the timeline visualization for team Purnululu.

However, we soon realized that the thematic coding done within NVivo could provide only a partial view of the ways in which micro-narratives operate within the broader structure of each team’s process due to the nature of its coding, which records information as individual and separate instances. To complete this picture, Author 3 coded and developed timelines, representing the full two-day period of the chats, creating an account of the process undertaken by groups and how different ways of interacting and thinking collaboratively were used by teams to ultimately create their knowledge product, including key moments and particularly notable elements or features of their interactions. See Fig 2 for a partial visualization of one of these timelines for team Purnululu. In conjunction with the coding done in NVivo, these timelines of the ways in which each team undertook completing its task enabled us also to more fully understand and represent the role micro-narratives played in their collaborative reasoning, as well as to understand other matters, such as the use of the SWARM platform’s lens kit tool, which is part of an ongoing research effort.

Simultaneously, we conducted a further series of meetings and discussions to clarify our model of micro-narratives and their role in the collaborative reasoning and knowledge production process in view of the various levels of coding and reflection we had undertaken to date, carefully comparing these with the emerging findings from the timeline coding and visualization process.

## Findings

### Micro-narratives

We observed that team members used plausible but incomplete stories, or micro-narratives, and compared the coherence, relevance and verisimilitude [1] of these as a means of informal hypothesis testing with regard to the evidence and intelligence they were given as part of the problem statement. These micro-narratives were weighed against each other, real world knowledge, and a virtual or ideal macro-level narrative which the team was constructing in order to understand and reason with the evidence presented in the problem.

This process can be clearly observed in the online chats, which provided the one “plenary” forum frequented by nearly all team members. These chat records provide a unique window into the manner in which narrative thought processes are contributing to the team’s reasoning and the attendant process of knowledge creation, a process that might otherwise be largely opaque beyond the team’s own report and any inferences that could be drawn from it.

We observed that often a very brief comment by an individual, or a conversational exchange between multiple team members, included multiple scenarios—some explicit and some implicit. Further, of those stated explicitly, some were only narrated in a fragmentary or disjointed manner. Participants used these micro-narratives to reason with evidence “on the fly”, allowing them to rapidly create and compare competing micro-narratives as possible best-fit solutions to various aspects of the problem or target of analysis, and in particular to cope with uncertainty and reason probabilistically about the micro-narratives.

In assessing the likelihood of the various suspects’ involvement in terrorist activity, participants were provided with a number of fictional intelligence sources. These included a laboratory report concerning the presence of the suspect’s DNA on a number of items: a computer keyboard, a mobile telephone and a soldering iron. Crucially, they were also presented with a summary of the forensic DNA techniques involved, and with potential issues with very small samples of DNA which have been “magnified” using PCR technology, a method that takes very small samples of DNA and replicates them thousands of times. Recent studies have shown that due to the small size of the samples, DNA can be transferred through interpersonal contact, potentially falsely placing someone at a location they have not visited. For example, following a handshake between two people, a person touching a knife may in some instances place the other’s DNA on that knife [34, 35]. The information about secondary DNA transfer was “hidden” within the larger discussion of forensic DNA techniques.

The possibility of secondary DNA transfer was a crucial one, and was designed to introduce uncertainty to the evaluation of evidence. In the problem statement, two of the suspects, the owner of the Telecom Shop (“The Owner”) in whose basement a bomb-making workshop was found, and an old friend of his (“The Old Friend”), had had dinner together the night prior to the raid on the shop. Both suspects stated that they shared a hookah pipe during that evening, increasing the odds of secondary DNA transfer between the two, as both may have repeatedly handled the same object over an extended period of time.

Both teams used micro-narratives in chats to assess and weigh a number of potential options that might explain the presence of the DNA on the items found, and the implications for how likely it was that either suspect was involved in terrorist activity.

For example, in the following two-minute chat exchange, Team Purnululu identifies a micro-narrative—DNA transfer occurred between individuals during a shared meal with a hookah pipe—and rapidly weighs this for verisimilitude and likelihood. The exchange of DNA by two individuals through sharing a hookah pipe would give a plausible explanation for the presence of the Old Friend’s DNA on an object (the soldering iron) in the bomb-making workshop discovered in the Telecom Shop’s basement. The team quickly also establishes another micro-narrative as being necessary: the Owner would have to have returned to the workshop after the meal in order to transfer his friend’s DNA onto the soldering iron:

**13:56 Kookaburra31:** I think it [the soldering iron] would be a standard repair tool for a mobile phone/ electrical device (but that’s my assumption!)
**13:57 Quoll97:** So really, the owners DNA could have been on there from ages ago. But still doesn’t explain how the old friend got his DNA stuck on there
**13:58 Platypus20:** If we consider the secondary DNA transfer, as I think someone mentioned previously, then the shared hookah pipe would explain how the old friend’s DNA got stuck on there.
**13:58 Kookaburra31:** Quoll97 There is a section in the background reading which talks about secondary DNA transfer. Seems unlikely, but worth considering.
**13:58 Quoll97:** But only if the owner went down into the workshop afterwards

In another example, Team Purnululu evaluates the intelligence presented to them in the problem regarding items found in the basement of the Telecom Shop, where the bomb-making workshop was found. The Owner has claimed that he rented the basement out on a cash basis to two foreign workers, and that he does not go there, while “The Courier”, the shop’s assistant and errand-runner, claims that he has never been in the basement. However, items that were formerly in the shop—a used mobile phone and a computer keyboard formerly used by customers—as well as the soldering iron found in the workshop, were found to have different constellations of the DNA of the Owner, Courier and Old Friend. Team Kakadu considers a number of micro-narratives in their assessment of this data:

**13:54 Currawong23:** Keyboard DNA could arise from secondary contact though, based off the amplification process.
**13:54 Crocodile97:** And the DNA for the keyboard has been explained as the old desktop from the office.
**13:54 Cockatoo27:** @Cassowary55- could the Courier’s DNA on the keyboard be from him using the keyboard legitimately in the shop/office?
**13:55 Bilby77:** That’s what I understood
**13:55 Echidna28:** Yeah they confirmed the computer used to be used for sales so that explains courier and owner’s DNA on it
**13:55 BlackCockatoo55:** The courier is also a store attendant—he works in the shop? He just doesn’t ’go that far out the back of the store building’.
**13:55 BlackSwan14:** Help me with something—if the owner/courier attests to not being in the basement for 6 months, how did the computer/keyboard/mobile phone/soldering iron end up in the basement?
**13:56 Echidna28:** The DNA of ‘others’ could also possibly be former staff? Other customers maybe. It’s difficult to say if there’s significance to that
**13:56 Cassowary55:** It could, it just never states that the courier used the computer in its old position
**13:56 Crocodile97:** @Blackswan!!! The computer was replaced a ‘few weeks ago’ who DID put it in the basement?
**13:56 Echidna28:** @BlackSwan14 That’s the thing. Whoever took them down there has to be involved
**13:57 Dingo48:** If not involved then at least aware of
[…]
**13:58 Currawong23:** While unlikely, it is possible that the items could have been sold to the ‘renters’ of the basement without any of the others actually going down there …
**13:59 BlackCockatoo55:** @BlackSwan14 they could have chucked the old keyboard out or put it out the back and the bombers took it for their basement?
**13:59 Cockatoo27:** Do the renters even exist?

In this exchange, it is evident that participants are engaging in what Bex et al. [36] refer to as generating stories and anchoring (or supporting) them with common-sense knowledge. In this case, for example, objects do not move themselves, and people who enter basements would generally notice a bomb-making workshop (although participants later question this latter point): in other words, participants are testing various scenarios for verisimilitude [1]. In this case, a participant’s question or statement may contain within it several implicit micro-narratives (such as the suggestion that the Courier moved the items and later lied about being in the basement, or that the Owner did so). This leads to several further possible micro-narratives:

1. The person who moved the items was involved in terrorist activities
2. The person who moved the items was aware of the terrorist activities but not involved in them
3. The computer was thrown out, and the renters recuperated it
4. The renters do not exist, and the Owner lied about their existence.

We found that the use of micro-narratives formed an integral part of both teams’ reasoning processes as they collectively analyzed the problem and went about producing a collaborative final report. Furthermore, as discussed in the following section, the conclusions reached by teams in their reports clearly indicate that the reasoning which informed the generation of micro-narratives was incorporated into the final knowledge product, the intelligence-style report.

### Micro-narratives, macro-narratives and the final report

We also analyzed the two teams’ final knowledge product—their submitted intelligence report. In particular, we looked for ways that the teams used the (“smaller”) conclusions reached through the use of micro-narratives in their chat deliberations to inform and produce the final report. Having observed the use of micro-narratives in our analysis of the chat transcripts, we hypothesized that groups would use these micro-narratives, as well as the way these fit together into larger macro-narrtives, to help construct their intelligence product,

Micro-narratives are not fully developed narratives, they are incomplete stories or scenarios. They serve as basic units that individuals and teams can debate, deliberate upon, and discuss in an iterative process in which micro-narratives are generated and weighed against each other for plausibility with regard to evidence, general knowledge about the world, and fit with other micro-narratives. They can then be organized and assembled into a larger, more developed narrative structure. This larger narrative structure would, for example, tell a story about each of the suspects in the Kalukistan problem, their interactions and their actions in the time period surrounding the bombing of the Ministry of Mines, and their participation in, knowledge of (or lack thereof) the bomb factory in the basement of the Telecom store. Because this full narrative(s) was never fully written down or recounted, but could clearly be inferred from the data in the chat transcripts and final report, we have chosen to call it a virtual, or ideal, macro-narrative. While aspects of this more complete story were found both in the chat transcripts and in the final report, this “macro-narrative” was never fully written down as one complete narrative—but elements of it could be seen in both the chats and the final reports. We could infer its existence as an “ideal”, or “virtual” narrative co-created by team members as they used the chat function, and we could also view “glimpses” of these macro-narratives in the final report.

In the exercise, the teams were directed to assess four different suspects in terms of their likely involvement in terrorist activities. We took each suspect’s assessed involvement, and its justification, to be a macro-narrative, as they were composed of relevant micro-narratives developed over the exercise justifying the assessment. Importantly, since the problem specifically asks for four separate assessments, each assessment was a natural candidate for conceptualization as a macro-level narrative.

In the previous section, we saw an example of how two teams engaged with the possibility of DNA transfer by creating micro-narratives to reason with evidence. The final reports of both teams synthesized these micro-narratives into a more complete narrative as part of their final report. Team Purnululu wrote:

The Old Friend’s DNA matched a sample collected from a soldering iron in the bomb workshop. We assess that transfer of The Old Friend’s DNA through contact with The Owner provides a plausible explanation for the presence of his DNA at the bomb-making site. We note that under test conditions, secondary DNA transfer was detected in 85% of cases following extended hand-to-hand contact, which could have occurred through The Owner and Old Friend sharing a hookah pipe the evening before the explosion. While the DNA transfer could have occurred in reverse, implicating The Old Friend, we assess that The Owner’s presence at Oasis Telecom immediately after their evening meeting and The Owner’s DNA on all three items found in the workshop indicate it is much more likely to have been transferred by The Owner. We assess that it is plausible that The Old Friend directly handled the soldering iron, but that his DNA was more likely to have been transferred in the absence of any other evidence linking him to the workshop.

Similarly, team Kakadu concluded that:

DNA material obtained from soldering iron could be explained by secondary DNA transfer: If a) the Old Friend and Owner sharing a hooker pipe the night before the bombing (or potentially at some earlier unidentified time) and b) the Owner touched the soldering iron that night or on the day of the bombing (or potentially at an earlier time).

**Fig 3:**
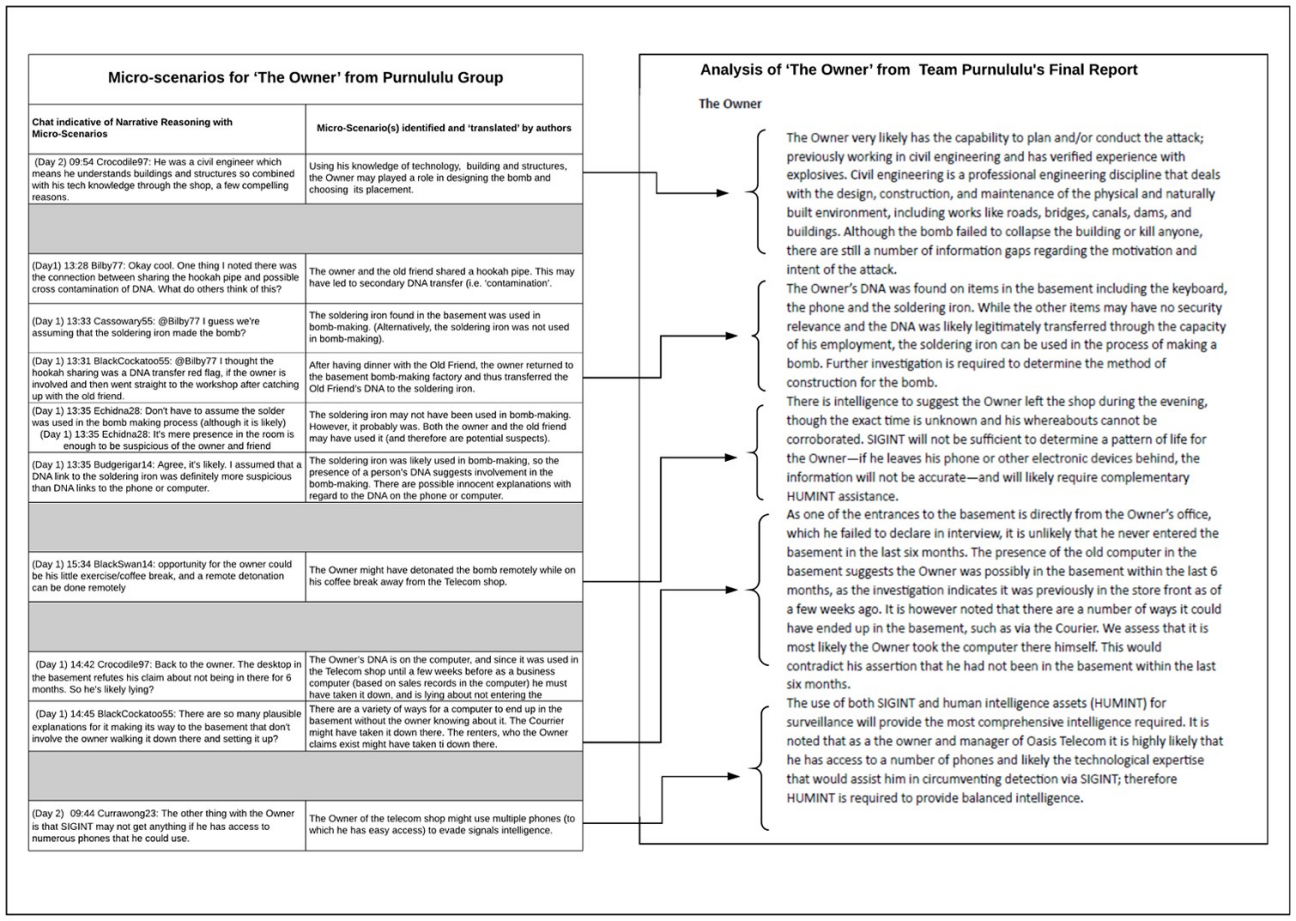
Micro-narratives and their relation to the final report.

In Fig 3 we present extracts from the chat transcripts of the Purnululu team, identify the micro-narrative which we have identified and “translated” from the transcript, and point to the macro-narrative section of the final intelligence report to which the micro-narrative corresponds. For brevity, we have selected only one macro-narrative, namely the team’s analysis and conclusions regarding the Owner.

**Fig 4:**
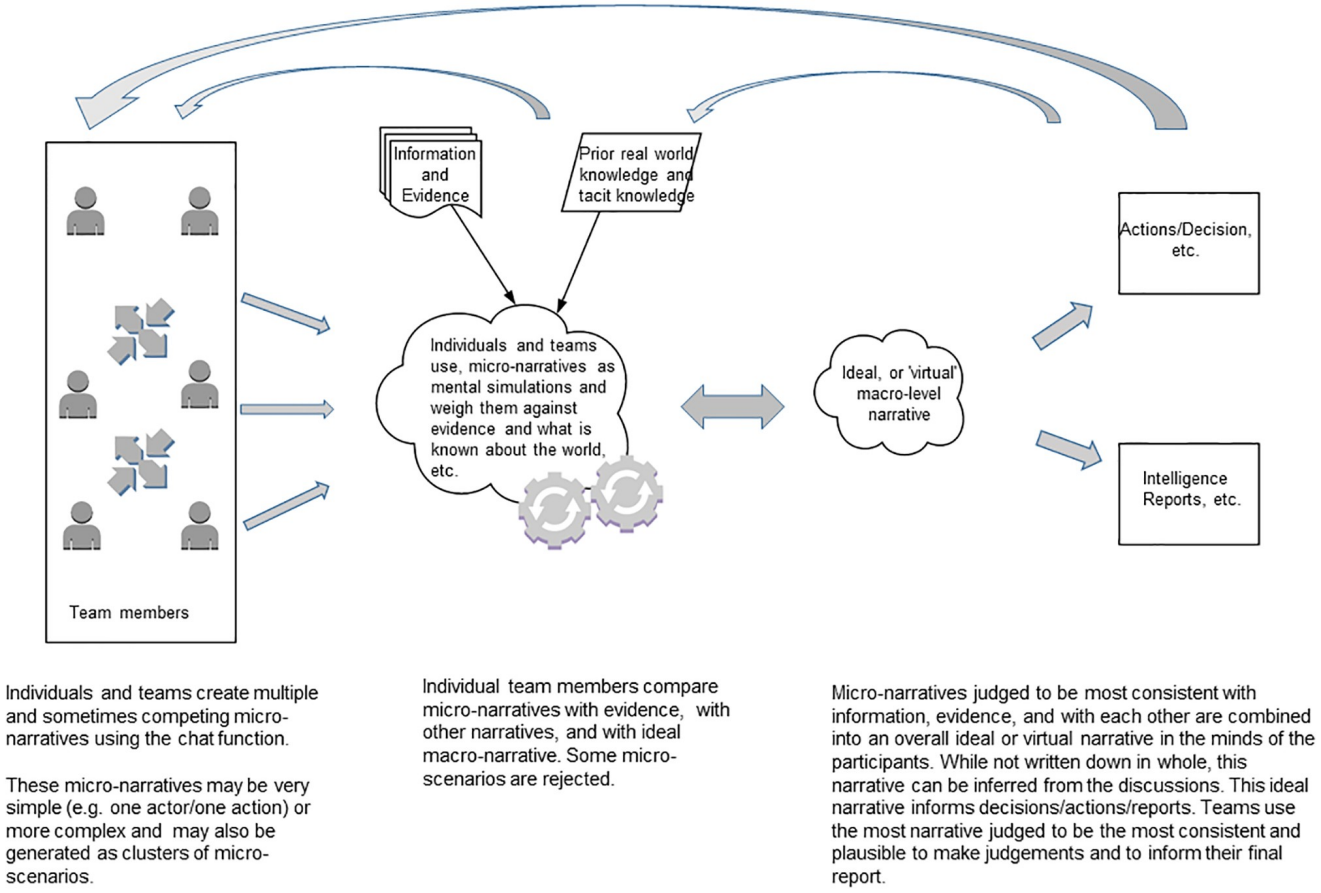
The process of micro-narrative generation and collaborative reasoning in producing judgements and the final intelligence report.

In Fig 4 we outline the process we observed participants to be following in their production of their final report: team members (pictured on the left) discuss the available data and the set task among themselves using the chat function of the SWARM platform, and in so doing generate multiple competing micro-narratives. These micro-narratives are weighed against one another, against the information and evidence available, and against existing implicit (and sometimes explicit) real-world knowledge (pictured in the center). We theorize that this occurs as team members run mental simulations, which they summarize and describe to others in the form of micro-narratives. Some of this sharing and discussion leads to a re-evaluation of suggested micro-narratives, as well as to new ones, which in turn leads to an additional “round” of mental simulation and both individual- and group-level consideration of existing micro-narratives. This gradually leads to the building up of those micro-narratives assessed to be most consistent with the evidence and with one another, leading towards an ideal, or ‘virtual’ narrative which can be inferred. Importantly, this process is not a linear one, but rather is gradual and iterative, with the constitutive stages being repeated and their contents altered as contending micro-narratives and their impact on the emerging micro-narrative(s) are assessed and discussed.

## Discussion

Telling stories, and using narrative, is a fundamental part of how we understand the world. According to Aristotle, the purpose of a story is to show us “what may happen—what is possible according to the laws of probability or necessity” [37]. Narrative is a cross-cultural human phenomenon, and may have evolved as a means to acquire and transfer information about the environment and to create shared, collective and public mental models of the world and reality [22, 38, 39].

Over the past few decades, researchers in the humanities, psychology and organizational studies have contributed to a growing body of interdisciplinary work connecting the study of narrative with our understanding of human society, the workings of the human mind, and our perception of the world. People use narratives and stories to compare perspectives, organize experiences, represent and “construct” reality and personal identities, elucidate goals, evaluate strategies, interpret results and outcomes, evaluate evidence and judge the validity of knowledge claims. [1, 4, 40, 41].

In the last several decades, narrative has been an expanding area of interdisciplinary research, and has inspired “research questions with direct relevance to national security” [42]. There has also been substantial and related discussion of the role of narrative as a mode of thinking and reasoning with evidence within the fields of cognitive psychology, jurisprudence, and security studies [1, 7, 43]. For example, Clark [7] and Heuer and Pherson [8] emphasize the role of narrative in producing scenarios for strategic business-planning, and their role in operational and tactical planning for military and intelligence organizations has also been explored extensively in the literature on intelligence analysis. Many of these research questions involve the emergence, understanding and influence of narratives on society and sections of societies (extremist networks, for example). In this paper, however, we are interested instead in the role of narrative in complex reasoning tasks and collaborative knowledge production in a complex, uncertain, and rapidly evolving world. This is the type of reasoning and knowledge production that intelligence analysts are routinely asked to produce.

Our findings support and contribute to the growing body of research supporting the simulation model of narrative. Mar and Oatley [19] argue that stories act as a kind of mental simulation of social interactions and of the minds of others, where, “like other simulations (e.g. computer models), fictional stories are informative in that they allow for prediction and explanation while revealing the underlying processes of what is being modeled.” These simulations allow social interactions, and other people who may be very different from ourselves, to be understood without being perceived directly [22]. Narratives also allow individuals to share these; they allow complex knowledge about the world to be transferred between multiple individuals in a readily comprehensible manner. This allows for simulations of the world to be shared and compared and for creation of collective and public mental models of the world and reality [22, 38, 39].

In the context of intelligence analysis, as also with scientific claims, it is not sufficient that a person or persons making a claim have some level of authority; rather, a judgement of validity must be based on weighing and evaluating the argument, evidence and “intel” put forward in support of a knowledge claim [41]. Reasoning with evidence, the exploration of alternative hypotheses, and testing or challenging assumptions are of fundamental importance to decision-making in uncertain and complex environments [7, 10, 44, 45].

We found that teams used micro-narratives to assist in reasoning with evidence, the exploration of competing and alternative hypotheses, in making judgements necessary to solve a difficult intelligence type problem and in the production of an intelligence report. We believe that future research can elaborate the role of narrative and narrative thought processes in collaborative knowledge-building and knowledge-building more broadly.

### Micro-narratives, narrative and analytic techniques

We have used the terms micro-narrative and micro-narrative to describe the often quite brief and fragmentary narratives which teams produced in the chat function of the SWARM platform. In doing so we are consciously drawing a parallel between the “on the fly”, informal reasoning process we observed as it emerged in the text chats on the SWARM platform, and the structured analytical technique of scenario generation as it has developed in post-WWII strategic thinking in the public and private sector particularly in the U.S. and France [46–48].

While scenario analysis continues to evolve, it has become an established structured analytical technique for predicting and considering multiple possible futures in intelligence analysis [8] and other fields, such as environmental assessment and modelling [49]. In business, scenarios are used for strategic planning often on a global scale, while military and intelligence organizations also employ them at the tactical and operational levels [7]. Scenarios can also be used to consider multiple possible past scenarios leading to the present, or “what really happened” [50]. What distinguishes the micro-narratives we observed being used from the structured analytic technique of scenario generation is their brief, fragmentary, and/or disjointed nature, as well as the informal context (text chat) and emergent, unstructured manner in which they are generated. In future research, however, we are interested in whether the use of micro-narratives, and narrative more generally, may serve as the basis for new structured analytic techniques, or may explicitly be integrated into existing processes and methods.

We are also particularly interested in developing tie-ins with related narrative techniques, such as what futurist and author August Cole refers to as FICINT [51], or Fictional Intelligence, in which fiction writing is combined with intelligence work to develop plausible scenarios that help intelligence analysts and strategists to challenge their own assumptions and to be better prepared for future developments.

## Conclusion

This case study was undertaken as part of ongoing exploratory research associated with the testing and development of the SWARM platform. The SWARM platform has offered a critical window, or metaphorical microscope, for peering into and better understanding a complex reasoning process that would otherwise be largely opaque. Using chat transcripts and final knowledge products in the form of an intelligence-type report, we observed that teams of individuals used micro-narratives and incomplete stories to make hypotheses about evidence, motivations, actors and actions, and so on. They then weighed these often competing scenarios against each other, the evidence, and their knowledge of the real world, and used these micro-narratives to create larger, more complete ideal narratives regarding the intelligence-type problem they were tasked to address. While these larger narratives were not written down, we could infer them from our analysis of the teams’ chat and the production of the final intelligence report. The team members then used these larger, more complete narratives (which often exist only ideally, or virtually, as co-created narratives in the minds of the team) to write an intelligence-style report evaluating the likelihood that each of the 4 suspects was involved in terrorist activities. We further inferred that the use of these micro-narratives in the creation of a larger, ideal narrative were indicative of a kind of mental simulation, consistent with models of narrative thought processes from the field of cognitive psychology (e.g. Mar and Oatley [19]).

While narrative has received increasing attention in a number of fields in recent years, scant attention has been paid to the role of narrative in complex problem-solving outside of the literature on jury decision-making (e.g. Pennington and Hastie [4]). We hope this paper will generate further discussion, while also contributing to empirical and conceptual research into the use of online collaborative platforms and collective intelligence generally, and to understanding the role of narrative and narrative thought processes in complex reasoning more specifically.

As organizations attempt to navigate, adapt to and coevolve with complex environments [52], the ability to rapidly process data and information into knowledge that can inform decision-making is critical [13]. The use of online collaborative platforms is changing the way individuals and teams work. Organizations are increasingly seeking to harness the “power of crowds” and collective intelligence in areas ranging from engineering and design to intelligence analysis by agencies tasked with domestic and national security. This paper contributes a fuller understanding of the role narrative can play in collaborative reasoning and knowledge creation, and we hope it can help point the way to further research into the importance of narrative to complex reasoning and problem-solving.
